# Potential Use of Community-Based Rapid Diagnostic Tests for Febrile Illnesses: Formative Research in Peru and Cambodia

**DOI:** 10.1371/journal.pntd.0007773

**Published:** 2019-10-28

**Authors:** Valerie A. Paz-Soldan, Amy C. Morrison, Heng Sopheab, Julia Schwarz, Karin M. Bauer, Jennie L. Mckenney, Chhorvann Chhea, Vonthanak Saphonn, Dyna Khuon, Robert D. Hontz, Pamina M. Gorbach

**Affiliations:** 1 Tulane School of Public Health and Tropical Medicine, New Orleans, LA, United States of America; 2 Universidad Peruana Cayetano Heredia, Lima, Peru; 3 Department of Pathology, Microbiology, and Immunology, School of Veterinary Medicine, University of California, Davis, California, United States of America; 4 U.S. Naval Medical Research Unit—6 (NAMRU-6), Lima, Peru; 5 School of Public Health, National Institute of Public Health, Phnom Penh, Cambodia; 6 Icahn School of Medicine at Mt Sinai, New York, New York, United States of America; 7 University of Washington, Seattle, Washington, United States of America; 8 University of California Fielding School of Public Health, Los Angeles, California, United States of America; 9 University of Health Sciences, Phnom Penh, Cambodia; 10 Naval Medical Research Center, Fort Detrick, Maryland, United States of America; George Washington University School of Medicine and Health Sciences, UNITED STATES

## Abstract

In 2012, the U.S. Defense Threat Reduction Agency Joint Science and Technology Office initiated a program to develop novel point-of-need diagnostic devices for surveillance of emerging infectious diseases including dengue, malaria, plague, and melioidosis. Prior to distribution of devices to observe their correct use among community members in Iquitos, Peru, and Phnom Penh, Cambodia, research was conducted to: 1) assess acceptability of use, including the motivation to use a rapid diagnostic test (RDT) before or instead of seeking care at a health facility, 2) explore comprehension of RDT use instructions, and 3) examine possible strategies for large scale RDT distribution and use at each site. In February 2014, 9 focus group discussions (FGD) with community members and 5 FGD with health professionals were conducted in Iquitos, and 9 FGD with community members and 9 in-depth interviews with health professionals in Phnom Penh. In both places, participants agreed to use the device themselves (involving finger prick) or could identify someone who could do so in their home or neighborhood. The main incentive to RDT use in both sites was the ability for device results to be used for care facilitation (post confirmatory tests), specifically reduced wait times to be seen or obtain a diagnosis. Comprehension of RDT use instructions was assessed in Iquitos by asking some participants to apply the device to research team members; after watching a short video, most steps were done correctly. In Phnom Penh, participants were asked to describe each step after reading the instructions; they struggled with comprehension. Health professionals’ main concerns in both sites were their community’s ability to accurately use the test, handle complicated instructions, and safety (i.e., disposal of lancets). Health system structure and ability to use home diagnostic devices varied in the two disease endemic sites, with substantial challenges in each, suggesting the need for different strategies for RDT large scale community use, and illustrating the value of formative research before deployment of novel technologies.

## Author summary

Development and use of devices to diagnose infectious diseases outside of health facilities (i.e., at home or in remote areas) continues to increase, providing new options for the follow up and treatment options of individuals, depending on the diseases. In this qualitative study, researchers in Iquitos, Peru, and Phnom Penh, Cambodia explored what local people thought about the possible availability of such a device to diagnose dengue and malaria in their own houses, and what would motivate them to use such a device instead of going directly to a health facility for the diagnosis. Participants reported being willing to use device on themselves and were motivated by the possibility that, by using this diagnostic device and taking the result to their health facility, they might be able to obtain quicker and more optimized attention at the health facility; for example, by obtaining a positive result to dengue or malaria on the rapid diagnostic device, they are aware they might still need confirmatory tests, but they would be able to reduce the wait time for obtaining a definitive diagnosis and starting treatment by a full day. Questions regarding accuracy of tests, complicated instructions and safety of using these devices in the community were brought up by participants—individuals living in the communities and local health professionals. Also, in this study, it was clear that if these devices were to be made available in Peru and Cambodia, different strategies for disseminating and using these in the communities would be needed to accommodate for different health infrastructure in both sites.

## Introduction

Recent pandemics of viral diseases such as Ebola and Zika have made clear the need for rapid identification of outbreaks to avoid widespread morbidity and mortality [1, 2]. Point-of-care (POC), or point-of-need (PON), rapid diagnostic tests (RDTs) have the potential to provide early identification of infectious diseases needed to launch rapid containment and prevention initiatives and avoid epidemics. A key characteristic of RDTs is the provision of rapid results that can guide clinical management of a disease [3]. Limiting the widespread application of RDTs, however are test reliability and performance, cost, regulatory approval, and clear integrated strategies for linking these results to treatment or patient follow up [3, 4]. Another possible limiting factor is the ability of individuals to apply RDTs to themselves or family members, which can depend on the complexity of the RDT procedures and the quality of the instructions, as well as individual-level factors.

Extending POC to PON implies moving the use of these devices from health facilities to households by either community health workers or residents themselves, with or without training on the devices. Home-based HIV testing has had widespread implementation and acceptability in part because of its inherent protection of patient privacy [5–7]. Likewise, malaria RDTs are now available commercially in many endemic countries and are used increasingly by community-health-workers in national malaria programs, especially in areas without access to laboratories [8–11]. In the case of malaria, there is a high rate of malaria treatment without testing (presumptive treatment), leading to ineffective or unnecessary treatments, and delayed therapy for other treatable febrile illness [11]–hence the value of the new malaria RDTs. Like malaria, dengue has a large number of potential differential diagnoses that have different treatments. An accurate positive diagnosis, including by RDTs, could greatly aid health care practitioners manage dengue cases. Other uses for RDTs could include the battlefield or remote posts for military, sentinel surveillance or outbreak response.

In 2012, the U.S. Defense Threat Reduction Agency Joint Science and Technology Office initiated a program to develop novel multiplex point-of-need (PON) diagnostics for surveillance of emerging infectious diseases. The first generation devices evaluated included ones that tested for dengue, malaria, plague, and melioidosis, integrated with a platform to automatically upload test results to a cloud-based biosurveillance system [12]. Evaluation in endemic countries was initiated in 2014 in sites in Thailand, Australia, Sierra Leone, Cambodia, and Peru to assess the use of these RDTs as close to the point-of-need as possible, including home-based testing by residents. Both Iquitos, Peru and Phnom Penh, Cambodia were included in the study because they are sites highly endemic for two of these diseases: dengue and malaria (Cambodia [13, 14], Peru [15–18]).

While there is high acceptability of malaria RDTs use on children by community health workers (CHWs)[19, 20], as well as among health professionals [21, 22], little is known about the acceptability of using RDTs by community members on themselves or their children. This study was conducted among community members and health professionals in Iquitos, Peru, and Phnom Penh, Cambodia to: 1) assess acceptability and willingness to use *prototype* RDT devices that included endemic malaria and dengue, with a focus on the acceptability of use of the device on family members, including minors, 2) explore comprehension of RDT use instructions, and 3) examine possible strategies for large scale community distribution RDT use for each site.

## Methods

### Study areas

As the first stage of a larger multi-center Defense Threat Reduction Agency (DTRA) sponsored study to evaluate two prototype RDT devices, in early 2014, we conducted Focus Group Discussions (FGD) in the Latin American city of Iquitos, Peru and South East (SE) Asian city of Phnom Penh, Cambodia (see Table 1). These sites were selected for their high dengue endemicity. Iquitos City (population ~470,000) [12, 23] located in the Peruvian Amazon rainforest, is geographically isolated and only accessible by plane or boat, and has been the site of several studies, primarily on dengue epidemiology, since 1999 by the University of California at Davis/U.S. Naval Medical Research Unit Six-Iquitos group (NAMRU-6) [17, 18, 24–28]. In Cambodia, recruitment was from a peri-urban area of Phnom Penh (~3–4 km from city center and from the Meanchey Referral Hospital) that included the communes of Deum Sleng, Deum Sleng1, Deum Chan, Deum Chan1, Kandal, and Kandal1 in Chbar Ampov1 with an approximate population of 20,000. Both sites were predominately low-income, urban, and crowded. In urban Iquitos, the population has easy access to government health facilities, which contrasts to the Phnom Penh sites that relied heavily on a network of community health workers.

**Table 1:** Summary of FGDs and IDIs conducted in Peru and Cambodia.

	FGD		IDI
	Number of FGDs	Number of Participants	Number of Participants
**Peru**			
Community members (in Proyecto Dengue study area)	5	45	-
Community members (outside Proyecto Dengue study area)	4	30	-
Doctors	1	6	-
Nurses	2	16	-
Nurses involved in febrile study	1	5	-
Medical students	1	14	-
**Cambodia**			
Community members	6	36	-
Village health workers	3	19	-
Fever investigation team	-	-	2
Health Center staff	-	-	2
National Malaria Center (CNM)	-	-	2
National Institute of Public Health (lab staff)	-	-	2
Private pharmacy staff	-	-	1
**Total**	**23**	**171**	**9**

### Study design

We used qualitative research methods with community members and health professionals in both sites; these included FGDs (both sites), and key informant in-depth interviews (IDI) in Cambodia only (see Table 1). A total of 171 individuals participated in the FGDs (Peru and Cambodia) and 9 in IDIs (Cambodia). Our primary research goal in both sites was to explore strategies to increase acceptability and widespread community use of the RDT prototypes. Local realities, customs, and existing health infrastructure (e.g., community health workers) were different, so despite initially using the same focus group guide questions, as qualitative exploration took place in both sites, the methods used to examine the different strategies diverged. In Peru, we had a larger research team, including senior researchers (VAPS, ACM), all fluent in Spanish, so the team could adapt to suggestions from FGD participants in real time. For example, participants suggested video instructions, and our team had the capability to develop a video before the next FGD, and then adapt to subsequent suggestions.

#### Peru

In Peru, 9 FGDs were conducted with community members in two groups. For group 1 (5 FGDs, n = 45), participants were recruited from neighborhoods that were part of ongoing longitudinal cohort studies and had a long history of participating in research projects. Group 2 (4 FGDs, n = 30) participants were recruited from neighborhoods where there was no history of research activities. Recruitment of all community members was done by convenience sampling, specifically for individuals responsible for their family’s health (i.e., the ones mostly likely to apply the device in Iquitos, usually mothers), from centrally located (Districts of Maynas, Punchana) selected urban neighborhoods (see [25] for detailed description) by door-to-door visits one to two hours prior to the FGD.

Another 5 FGDs were conducted with health professionals and medical students (total participants: 41). Verbal informed consent was obtained from all participants. Detailed notes by two team members and audio-recordings were taken of all FGD discussions. The same evening, the research team reviewed and compiled all notes into a single document; although recordings were not transcribed (sound quality was inconsistent and, in a few, impossible to hear), audios were used to ensure the notes and possible quotes were complete and accurate (i.e., if quotes were not consistent among note takers). The FGDs were all conducted and analyzed in Spanish.

#### Cambodia

Six FGDs were conducted with community members and three with village health workers (9 FGD, n = 55). Additionally, key informant IDIs were conducted with health professionals (n = 9). FGDs were arranged among community members and village health workers within their communities with support and coordination from the National Malaria Center (CNM) and Naval Medical Research Unit-2 (NAMRU-2). In Cambodia, we coordinated with village chiefs who had information sheets to aid with recruitment of eligible community members 1–2 days prior to the FGD. Interviews with health workers were arranged with their respective institutions by appointment. All FGDs and IDIs were digitally recorded and then transcribed and translated, with verbal informed consent from all participants.

### Study procedures

FGD and interview guides were developed by the study team and translated into Spanish and Khmer for each site. Our primary interest was assessing community and health sector acceptability toward the use of two novel PON RDT prototype devices developed for the Department of Defense under the 24-month Challenge Initiative. The two devices were going to be subsequently evaluated for technical parameters (sensitivity/specificity), and study sponsors wanted to understand potential barriers to community members using these devices in their homes and what would motivate them to do so. Two RDT prototypes were used: one that detects dengue virus (DENV) and *B*. *pseudomallei*, and another that identifies DENV, *Plasmodium vivax*, *P*. *falciparum*, *Yersenia pestis*, and *B*. *pseudomallei*. Both required a few drops of blood from a finger-stick, so that people using the tests would need to use a retractable lancet and be provided basic biosafety materials and instructions to conduct the tests safely. Therefore, during FGDs, facilitators explained that the devices would not tell them specifically what disease they had (i.e., they would not know what the lines stood for), only that they would see a line indicating they had “something” requiring follow up. Results could not be given because these test devices had not been validated and it would be important that the results be evaluated by a trained health professional, so for the context of the discussions, we made it clear that a link to patient follow up or treatment have to follow use of the device.

#### FGD with community members

We initiated FGDs with an overview of a proposed study. To introduce the concept of an RDT, we asked participants about their familiarity and use of home pregnancy tests. We asked participants about diagnosing fevers and their use of thermometers. We then explained how rapid tests are being developed to diagnose causes of fever and that these devices could potentially be used by anybody at home. The prototype RDT devices (see description above) were shown to participants, followed by a series of hypothetical questions related to their interest in using them. No one left with an RDT to use at home.

**Fig 1:**
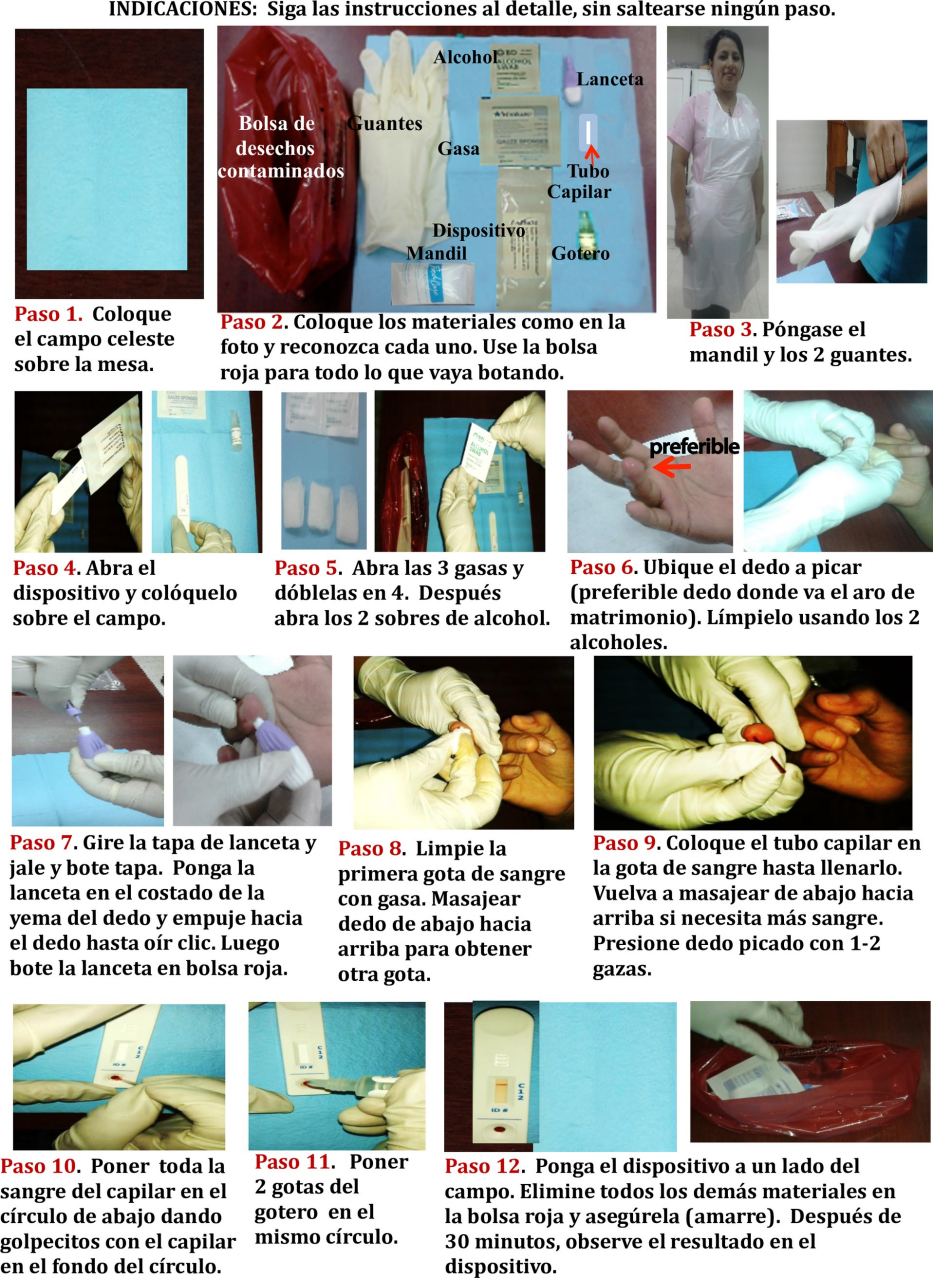
Written instructions in Spanish that were provided to focus group participants in Peru.

**Fig 2:**
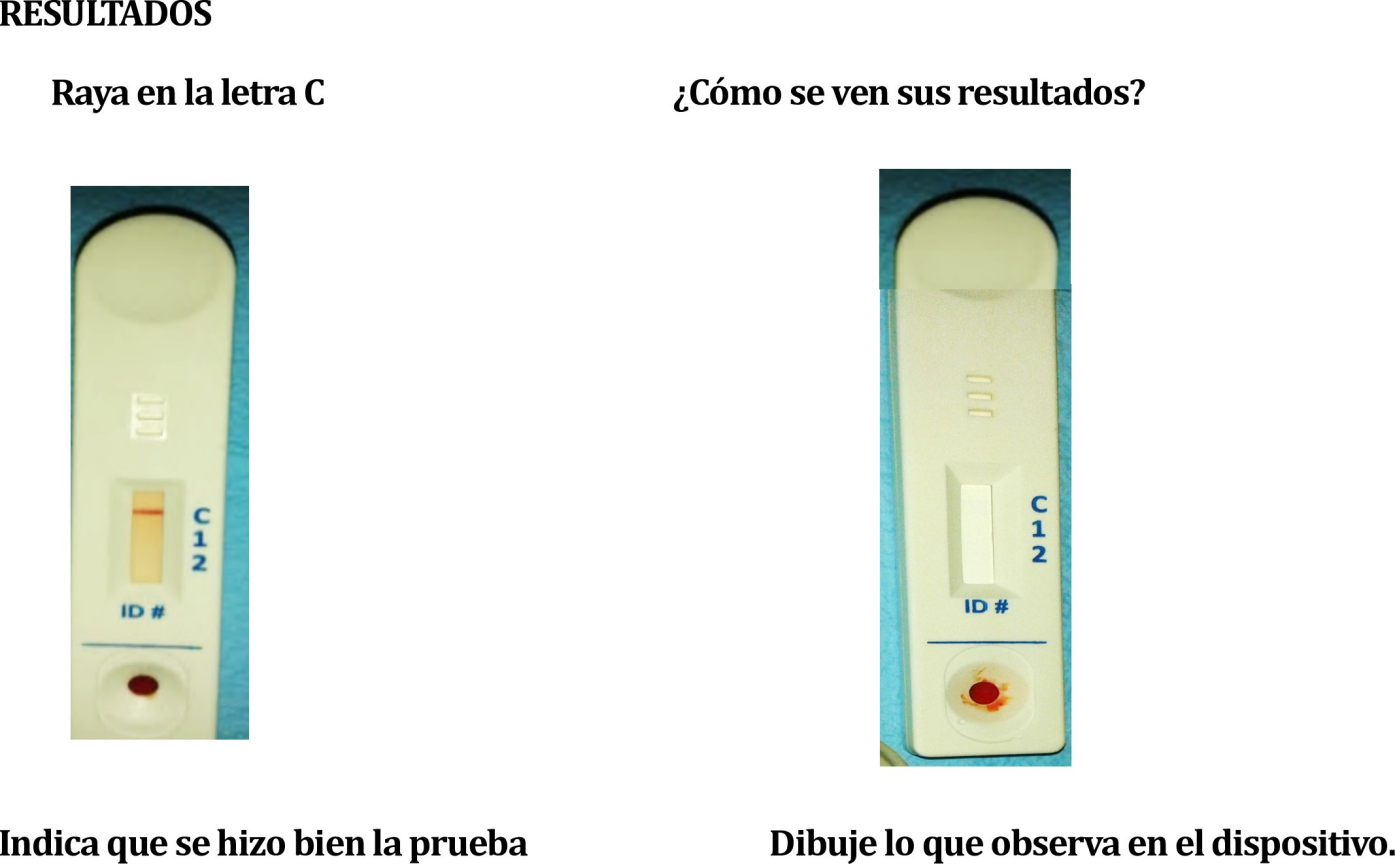
Preference for documenting observed results on paper based on what focus group participants viewed on rapid diagnostic test, in Spanish.

In the Peru FGDs, we then asked for volunteers to try and use the device, by breaking the participants into two groups: in each group, one FGD participant applied the RDT and another participant guided the person applying the RDT, while facilitators (ACM, JC, ER, AV, LQ) acted as patients. During the first two FGD, only paper instructions (step by step photos and text for each image) were provided (see Figs 1 and 2); in subsequent groups, an instructional video was developed by the research team so that participants could observe the procedure being conducted step by step. After each FGD, modifications were made to the video based on observations of this activity; this process allowed the research team to evaluate different ways to convey instructions and assess what worked best. Participants’ ability to use the device correctly, preferences for documenting observed results (Fig 2), questions, and reactions about the use of the device were recorded by the study personnel throughout the activity.

**Fig 3:**
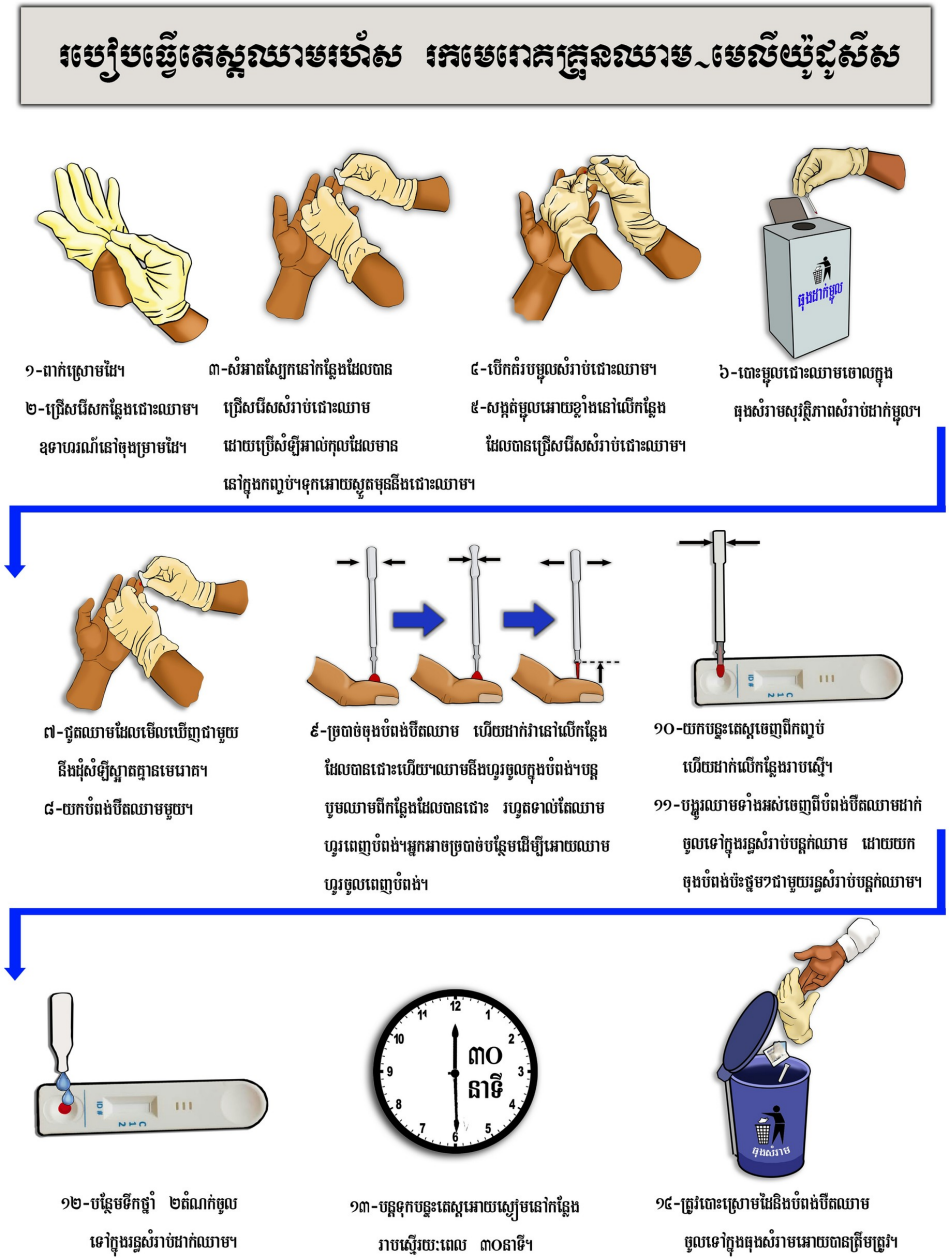
Written instruction in Khmer and demonstration of the test kit in Cambodia.

In Cambodia, a detailed instruction guide with images produced by a local artist for low literacy comprehension was developed and used instead of the video (Fig 3). Prior to testing their comprehension regarding using the device, community members were given an overview of the purpose of the study, then the device was passed around and described, with participants’ questions and comments recorded. FGD participants were provided with the device along with the instructions, given several minutes to read the instructions, and the facilitator then asked questions to assess their comprehension of the testing procedure and confidence in their ability to use the device. The research team demonstrated how to use the device on themselves and probed further on acceptability of the device in their communities and confidence in use.

Finally, in both sites, participants were asked about the acceptability towards the device, its possible use with family members, and what would motivate them to use the devices, and if they had any concerns about using the devices at home.

#### FGDs with health professionals in Peru

FGDs were initiated by polling health professionals from the public sector about their familiarity with RDTs. As with community members, participants were shown a video on how to use the RDT and asked to form teams to apply the test on facilitators; their responses, questions, and comments were recorded as described above. The discussion was then guided toward how the RDTs, including home use by community members, could be integrated into the current health system, and concerns about the RDT and its potential for integration into a local surveillance system.

#### IDIs with cambodian health professionals

Key informant IDIs were conducted in the participant’s workplace by two interviewers with prior appointment. One interviewer asked the questions while another took notes. The IDI guide covered the same topics as those in the FGD for health professionals in Peru (see above), but it was difficult to convene all health professionals at the same time.

### Data management and analysis

Data were analyzed in both countries manually (without the use of qualitative software), examining trends and findings for the pre-established themes (our main codes) in both countries. There were some topics that were not covered in both countries because implementation strategies diverged in both countries based on cultural context and health care resources available in each. Relevant quotes are used to exemplify these main themes and are presented in text.

### Ethical considerations and consent

The study protocol was approved by the Institutional Review Boards (IRBs) of Tulane School of Public Health and Tropical Medicine (545776–1), University of California at Los Angeles (14–000620), Naval Medical Research Unit Six (Protocol #NAMRU6.2014.0002) which included Peruvian representation, in compliance with US Federal and Peruvian regulations governing the protection of human subjects, and the Cambodia National Ethics Committee for Health Research (NECHR 0267). The protocol was also reviewed and approved by the Loreto Regional Health Department which oversees health research Iquitos. Since the only identifier to participation in this qualitative study would have been the signatures on the consent forms, the IRBs approved that all participants provide oral consent to participate in the focus group discussions (Peru and Cambodia) or in-depth interviews (only done in Cambodia), and to be audiotaped. After the consent form was read out loud in the focus groups and interviews, the researcher asked each individual for their verbal agreement to participate in, and be audiotaped for, the study, before initiating the data collection.

## Results

### Socio-demographic profile of the participants

In Peru, we principally recruited women (mean age 35 years, range 18–60 years) because they are generally responsible for health care for family members (females: 93%, 70 of 75 FGD participants) (Table 2; data only available for Peru). Participants had an average two children, of which the average age of the youngest child (in some cases this was a niece, nephew, or grandchild) living in the home of 4.5 years. The five FGDs (n = 41) with health professionals included: (1) two groups of nurses/nurse technicians and one group of physicians working in the febrile triage section of Ministry of Health facilities; (2) nurse technicians on cohort study research staff; and (3) a group of medical residents on rotation in local hospitals.

**Table 2:** Summary of demographic characteristics of people participating in the community focus group discussions in Iquitos, Peru.

FG N°	N° participants	Sex		Age Range	Knows how to use a thermometer	Would use the test on themselves	Would you use the test on a family member who is a minor	Estimated price participants would pay for a device (in US$)
		F	M					
1	10	10	0	27–45	2	10	7	--
2	10	6	4	18–60	0	6	10	1.75
3	8	8	0	23–37	0	7	6	1.75
4	9	9	0	30–48	2	8	9	1.75
5	8	8	0	22–40	2	8	7	1.75
6	8	8	0	26–54	1	7	8	1.75
7	6	5	1	33–52	--	6	6	1.75
8	8	8	0	20–44	0	3	2	1.75
9	8	8	0	22–42	--	8	8	--
**Total**	**75**	**70**	**5**		**7**	**63**	**63**	

In Cambodia, the mean age of male community FGD participants was 55 years (n = 32); many reported taking care of their grandchildren at home. In contrast, female community member participants (n = 23) were younger (mean = 40 years) and most were mothers (90%). The mean age of Village health worker FGD participants was 54 years.

The key informant IDI were conducted with health workers (n = 4), surveillance officers (n = 1), epidemiologists (n = 1), laboratory staff from the NIPH (n = 2), and pharmacy staff (n = 1)–representing staff from the health centers, a local pharmacy, referral hospital, National Malaria Center, and National Institute of Public Health. The key informants were predominantly male (89%, n = 8).

### Current practices of fever detection and management

In both Peru and Cambodia, few community members reported having a thermometer in their home and those who did, most did not know how to use it. The majority of participants described using touch to diagnose fever in their children, as well as identifying other symptoms associated with fevers, including shiny or glazed-look in eyes, rouged cheeks, lethargic behavior.

When participants in Peru felt fever required professional evaluation, the majority stated they would prefer to bring their child to a hospital instead of a health post (a lower level health facility), since health posts had long waiting times and were not well equipped: “*We don’t go to the health post*. *They attend us very late”* (FGD Iquitos, inside study zone).

In Cambodia, nearly all people reported bringing their febrile children to be checked by the Village Malaria Workers (1–2 per village) or to the nearest health center. As part of the Cambodian National Malaria Program, village malaria workers (who are usually the heads of the villages) record and report children with fever (>37.5 C) to health surveillance teams (called Fever Investigator Team) who then take blood samples for rapid testing and refer children to health facilities if necessary. Some parents reported taking their febrile children directly to health facilities because they worried about giving medicine to their children without a doctor’s consent: *“We could not give any [not prescribed] medicine to them since they are children*. *We could not give medicine prescribed by [seller] at pharmacy*. *We need to go first to see doctor*, *then [he/she] prescribes the drug”* (FGD Deum Chan). Some health workers reported that people self-medicated with medicine purchased at pharmacies or visited private clinics, where they felt services were friendlier and faster.

### Experience using other home-based RDTs

To assess familiarity with similar commercially available “rapid test devices”, we asked about home pregnancy tests. Most community participants in Peru, both inside and outside the study zone, were familiar with home pregnancy tests, and some could describe generally how to use them, but only few people had used or knew someone who used one. Most of the community participants in Cambodia reported no previous exposure at-home rapid tests. In contrast, the health professionals in both Peru and Cambodia knew about both pregnancy and other RDTs.

### Motivator to using RDTs: Reduce waiting time for diagnosis

In Peru, although FGD community participants had not seen any commercially available at-home diagnostic devices, enthusiasm for such a hypothetical febrile diagnostic product to use at home was nearly unanimous. The perceived reason was to avoid long waits at health facilities and to reduce waiting time for a diagnosis: “*It would be good [to use the RDT] to avoid going to the health post*. *With this [test] you can see a doctor more quickly without having to stand in line*” (FGD, Iquitos, inside study zone). Although no system is currently in place, participants liked the idea that having a diagnosis in their hand could facilitate or speed up their care once they presented at a health facility. Participants also felt that doctors and providers would take the patient more seriously because a positive test would mean that they had “something” for the health facility to follow up immediately and that this would facilitate their clinical evaluations: “*I like the test because… I would be able to obtain something*” (FGD, Iquitos, inside study zone).

Feedback from health professionals in Peru was consistent with that of community members, specifically that having patients arrive with a completed RDT would help them “streamline” the patient within the system. They did not feel that it would change the quality of the treatment, however, but felt that positive RDT result combined a clinical evaluation would increase confidence in a clinical management plan.

In Cambodia, the ability to detect dengue fever also made the test attractive to community participants, particularly since they had been exposed to annual dengue outbreaks. A quick result was appreciated by both community members and health workers. It was proposed that the test could be used as a first screening tool when a child has a fever, especially in remote and rural areas where access to health facilities is limited: *“If there is a test at home*, *we could test*. *If it is found that the child has dengue fever*, *then we can bring him to hospital*. *It saves a lot of time”* (FGD, Kandal village). It was noted, however, that it might not be of much benefit in urban areas where there is access to standard blood tests with modern laboratory instruments and equipment.

### Concerns about using RDTs ranged from wanting test results to quality of result based on user

Community participants in both sites questioned using the test if they did not get a result, or, at minimum, were alerted that they had something that requires action, regardless of how simple or non-invasive the test procedure was: *“If [we don’t know the result] after testing*, *we don’t know what we test for”* (FGD, Deum Sleng village). Participants wanted to receive results eventually, there was little interest in using the devices, if the results would only go to central location (i.e., Ministry of Health for surveillance). Participants in both sites understood and accepted the idea of a confirmatory laboratory test following a positive RDT, which is consistent with their standard operating procedures and with current WHO recommendations for dengue fever; they also understood that a negative test result may not always be accurate. Assuming a hypothetical streamlined process for getting health care once they had a result, all of the respondents accepted that they would need to take the device to a health facility for a professional to interpret the results, conduct a confirmatory test, and recommend treatment: *“We don’t have the expertise to know what to make of the result” (FGD Iquitos*, *outside study area)*.

A major concern of health professionals in both settings was that, in the context of limited diagnostic resources at the health facility level, it was wasteful to have these RDTs in patient’s homes if they needed to seek diagnosis and care at the health facility anyway. Furthermore, they felt that since confirmatory tests were still required, it made more sense that the RDTs be applied at health facilities. Both health professionals and medical students, in Peru expressed concern about the quality RDT test results when applied by untrained people: *“We will find people of different levels and they won’t be able to use the test”* (FGD with nurses in Iquitos). They saw the value of a home-based test and proposed a clinical management strategy, but expressed concerned about community members *“using the test correctly”* and about wasting tests when there was not a real possibility of dengue fever.

The quality, reliability, and sensitivity of the RDT were questionable to health workers in both Peru and Cambodia, and hence a concern, but those in Cambodia noted that the device still has benefits: *“RDT is better since it gives a faster result*. *However*, *many years’ experience show that sensitivity is questionable*. *Still*, *it is preferable for many users”* (IDI, NIPH lab staff, Phnom Penh). In Cambodia, concerns about false positives were also expressed, as well as questions regarding RDT maintenance in hot and humid weather: “*From my experience working in the malaria program*, *similar test kits faced errors due to inappropriate storage of the tests”* (IDI, Malaria officer, Phnom Penh).

### Acceptability for applying the RDTs to adults or minors of the home was high

After applying or observing application of the RDTs, all Iquitos participants expressed confidence performing the test on themselves: *“I would do it on myself if they gave me the instructions”* (FGD Iquitos, inside study zone). They were also confident about letting a family or a friend conduct the test on their child. When asked directly about whether or not they would be *willing* to use the test on a child, we observed that those from Iquitos research study zones reported being more willing to use the test, compared to those outside study zones. Regarding pharmacy-based testing, there was hesitation because of a potential cost for testing or that the pharmacy staff would not be trained properly. Although the research team was exploring the possibility and strategies for free distribution of RDTs, when asked about what they would be willing to pay for such RDT, the majority said 5 soles (or USD 1.75) would be right, and even 10 soles would be too high. In Iquitos neighborhoods with ongoing dengue research, some responded that they would *“wait for [study staff] from NAMRU”*, the local surveillance program supported by NAMRU-6, rather than go to a pharmacy.

### Comprehension of RDT use instructions: Community members felt they could use it, and in Iquitos did so very well, but health professionals in both sites expressed some skepticism about community member use

In Peru, participants were split into two groups per FGD, and applied the RDT to a research team member after reading the instructions and watching a short instructional video (in only 7 FGDs). In all groups, there was one person reading out the instructions, and one person applying the device, while our research team observed. In all groups, except for limited coaching by the research team on massaging a finger more thoroughly to get a drop of blood or using the pipette correctly, FGD participants were able to apply the test successfully. All groups in Peru unanimously agreed device use steps needed to be simplified, particularly if one person applies the device by him or herself, noting it would be easier to apply the device with another person reading the instructions, as they had done in the group.

After reviewing the test kit instructions and watching the research team apply it to themselves, Cambodian participants felt that they could master the use of the test kits even though they had never used something similar before, and reported feeling confident that others in their community could use it: *“[All participants]*, *there is no problem*, *[using the test] is easy*, *so [other people in community] could also use it like us”* (FGD, Deum Chan village). They felt the procedures were simple, requiring only a few drops of blood: *“[The lancet] is easy to use*. *We only press little bit*, *the blood comes out”* (FGD, Kandal village). The finger prick method was also preferable to drawing blood from vein using needle and syringes, especially for children.

The views from the health professionals in both countries were consistently different from the community members. They believed that the process was not easy for non-health professionals, advising that frequent training and monitoring would be needed: *I think it is very difficult even for health workers who are rarely exposed to such work [using a test] and may be still hard except for the lab personnel…*. *handling the pipette is not easy”* (IDI with surveillance officer, Phnom Penh). Most expressed it would not easy for community members to perform the test by themselves, especially using the lancet to do the finger prick and the pipette to draw drops of blood. They recommended lancet types that would be easier for lay people to use.

### Complete use kits must accompany the RDT, including disposal instructions

Participants from Iquitos stated unanimously that the RDTs must be part of a complete kit, that includes required materials (gloves, lancets, chuck pads, gauze, bandaids, etc.) written instructions, a phone number to call for assistance: “*Give all the materials in a packet at one time instead of asking them to purchase each part separately”* (FGD with community members inside study areas in Iquitos). This was reiterated in Cambodia, since the supplies required were unlikely to be available in their homes. In Peru, participants suggested clear packaging, making it easy to see what was inside.

In Peru, questions emerged about disposal of used packaging, particularly lancets; participants suggested taking used equipment to the health facility when going to obtain test results or confirmatory testing: *“The bag should have information about how to eliminate it*, *if they should take the waste to the health center”* (FGD with nurses in Iquitos).

Several Peruvian mothers stated they would not use the gloves with their children because “*it wouldn’t bother them”*. Health professionals expressed biosafety concerns, especially Hepatitis B, emphasizing the importance of biosafety had to be included in device instructions. These issues did not come up in the Cambodian FGDs.

Regarding strategies for community-wide distribution of devices, participants in Iquitos suggested distributing them in areas with recent transmission: *“[It should be done] like how fumigation is done in the sector where dengue cases appear*, *then it would be good to distribute this so that the people can use the RDTs”* (FGD with community members outside of study zone in Iquitos). The discussion regarding the preferred device distribution method in Cambodia focused on whether the test kits were to be sold or provided for free. Community members and health professionals reported that the pharmacy was the most acceptable site for selling the RDT kits: *“[The test kits should be sold] at the pharmacy because if we need medicine we could also buy the test kits”* (FGD, Deum Chan village). In both sites, health professionals recommended promoting new RDTs through a national program to enhance people’s confidence and trust in the new product.

### Written and video instructions should be included in RDT package

The participants who viewed an instructional video in Peru were observed to be more confident and proficient in applying the device compared to those only using written information: *“With the instructions I can do it*, *but with the video it is better”* (FGD with community members inside study zone in Iquitos). We observed that those who saw the video prior to device use only referred to written instructions as a *reminder* of the steps versus others who depended exclusively on the written instructions for each step. Participants mentioned that illiterate users would need very clear picture instructions.

In Cambodia, one idea that emerged was that written instructions could also be in English because it would be seen as a foreign product, which tends to have more prestige. Other suggestions focused on readability, specifically use of short and simple words, as well as large font size.

### Need for development of a better result-sharing system with health professionals

The results produced by the device need to be checked and interpreted approximately 15–20 minutes after being used. Realizing that this could pose difficulties for some families anywhere (e.g., the test might specifically be used outside of regular health post hours), in the Iquitos FGD we discussed sending a photo image of the test result to a health professional via cell phone. Fewer than half of each 8–10 person FGD participants had cell phones, of which only 2–3 per group had cameras; only one person had ever used a cell phone to send a photo. Despite this, FGD participants felt a cell phone image was a feasible means to document test results in Iquitos. As an alternative, we asked Iquitos participants to look at pictures of RDTs with red lines representing different results, and follow instructions to either: 1) draw in red lines on the picture of an empty device, or 2) select the identical image from a series of pictures of the device depicting different results (see Fig 2). We observed participants making mistakes using both options, but participants made less mistakes, and also preferred, the first option; however, it is clear that training users on how to interpret and document the RDT results is a critical step that requires further consideration.

The preference for handling, interpreting, and reporting results in Cambodia was to use the health center staff rather than health village volunteers. Most of the health center workers were able to use and send text messages or emails, allowing for quick data management and reporting: *“I am confident in sending the code through email… It is only a matter of click and send”* (IDI, health worker at Meanchey RH). However, this option would work if the health village volunteers could send a picture of the RDT showing a result, and receive a call or text with a response. Again, strategies for distribution and for obtaining results will vary per site, and this supports the notion that considerations for RDT availability need to be locally considered and culturally appropriate. The option of taking the RDT to a health facility could also be problematic with some RDTs if the results need to be interpreted at the 20 minutes time point. As was the case with Iquitos, other solutions need to be explored for helping users interpret and/or document results.

### Distribution of RDTs needs to be coupled with outreach and training

All Iquitos and Phnom Penh FGD community participants felt the device distribution needs to be coupled with strong community outreach and training. Study participants strongly believed that acceptability would be high due to the simplicity of the test kits. However, the actual use of the test would rely on the accuracy of its result: *“[You] need to give the test kits to population to try it*, *if it is accurate*, *then they will choose the test kits”* (FGD, Deum Chan village).

Iquitos and Phnom Penh health professionals and medical residents/students suggested various awareness-building activities regarding RDT procedures and use: teaching during medical consults, training in health centers, house-to-house visits for one-on-one training, community presentations, and public service announcements: *“The hospitals and health centers should train patients [on RDT use] during appointments*. *And the patients will do the same for their families so that it is multiplicative”* (FGD with medical residents in Iquitos). It was inconceivable that the tests would be distributed without outreach. Cambodian participants also suggested that the Village Heads or village health workers could be trained as a resource or trainer if the test kits were introduced to the communities.

## Discussion

This qualitative study represents the first step in the potential implementation of RDTs for febrile illnesses, in particular dengue. There was interest for at-home RDTs in Iquitos, Peru and Phnom Penh, Cambodia, consistent with the acceptability of similar RDTs in other locations, particularly for malaria [21, 22]. This study found a range in the ability of community members to apply the RDTs on their own. There are many alternative use strategies for RDTs that might be more effective than direct distribution or commercial availability to community members, especially in locations such as our Cambodia site that had a functional network of community health workers–these need to be explored by site. Community member RDT use competency, effectiveness of instructional materials, and result reporting are all significant challenges that require further investigation before widespread use of RDTs for home use can be implemented. Successful implementation of RDTs for research or public health purposes requires subsequent testing–observation of competency testing, development of optimized health services (a system for test users to get clinical follow up), and acceptability of test use with approved devices.

Studies in sub-Saharan Africa and Southeast Asia have demonstrated that clear, simple instructions increase the ability of community health workers to use malaria RDTs and interpret the results correctly, rather than relying solely on manufacturer instructions [29, 30]. Potential devices need to be simplified as much as possible, and their distribution must be as part of complete kits that include all the material necessary to apply the tests with appropriate biosafety, and instructional materials balance sufficient detail without overwhelming the user. Both written and video instructions should be provided with the device package if possible. Additionally, substantial community awareness and education campaigns need to be conducted to ensure that the population is prepared to apply the device correctly.

The main motivator driving acceptability to use the device by community participants in both sites was to streamline and reduce waiting time in health facilities. Both community members and health professionals thought using RDTs as part of “fast-track” program in the current health care system was very appealing, despite a clear understanding that confirmatory testing would still be required. The perception was that people presenting with a preliminary result, would facilitate subsequent testing. In Phnom Penh FGDs saw more value in using the tests in rural areas far from health facilities (compared to urban areas), proposing their application by village health workers or Village Heads.

A strong link to follow-up with the health system is critical in both countries if RDTs are to be distributed at the community level, and health facilities should be prepared for an influx of questions regarding the device. A critical issue will be the ability of community members to read the test directly or find an easy way to document the result (i.e., photo); this is particularly relevant for RDTs where results are only valid during a specified time window (i.e., 30 minutes after adding blood and buffer). If the test must be read at a health center, rather than providing a positive or negative result, there was consensus that it is more practical to do the test at a health facility. Despite concern from the manufacturers that patients would not seek care if results were negative, participants in both countries understood the concept of confirmatory testing, regardless of test result. And they clearly understood that if they could see a positive result, this would validate their need to seek health care from an expert.

Community wide distribution and implementation of RDTs requires careful consideration of who would administer the tests and if they should have training or rely exclusively on written or video instruction, and of how RDTs will be distributed (i.e., through community volunteers, commercial locations, at health facilities, or distributed by health officials); decisions regarding the who and how will depend upon local health systems structures and resources. Studies have shown that individuals have accepted distribution by Community Health Workers (CHWs) or even community medicine distributors where they exist [19, 20]. In Cambodia, the Village Health Workers were mentioned numerous times, but because they were not present urban Iquitos, not discussed in the Peru FGDs. Health professionals did discuss CHWs in rural communities, but expressed concerns because of high turnover. Moreover, in Cambodia, the discussion on the system of distribution depended on whether the tests would be free (preferred distribution at households or health centers) or sold (pharmacies preferred). A study in Nigeria showed that follow-up SMS text messages increased adherence to malaria RDT results for patients that purchased tests at privately-owned drug retailers [31]. Regardless of approach for distribution and result provision, integration into existing health systems will be critical the use of RDTs outside health facilities.

A critical question associated with the use of RDTs in Public Health programs is clinical follow up, which depends on treatments available, best illustrated by contrasting dengue with malaria infections. Chemotherapeutics are available for malaria, so an accurate result could greatly enhance access to treatment. Previous studies have shown that health workers may treat a patient regardless of their negative result on malaria RDTs, at times leading to the unnecessary prescription of anti-malarial drugs [32]. Thus, clear guidelines accompany RDT use by CHWs (or community members) especially if test results are negative; these guidelines should provide clear guidance on patient interaction, alternative causes of disease, and support for building health worker-patient relationships [33]. This argues that RDT use in these settings be accompanied by training, rather than product instructions/educational materials.

A major concern expressed by both Peruvian and Cambodia health professionals was that RDTs in the hands of community members would waste resources unnecessarily, particularly because government health facilities often lack the financial resources for diagnosis. Using RDTs will vary with the pathogen of interest (how results link to clinical management or therapeutics), sentinel surveillance, outbreak response (i.e. quarantine, or targeting of vector control), strength of local health infrastructure to adequately incorporate RDTs in to their programs. Additionally, concerns about misuse or improper interpretation of RDT results were high, especially because RDTs sensitivity/specificity specifications can vary dramatically under field conditions (health clinics). How will people respond to a negative RDT result? If a negative result delays or alters seeking health care, there could be serious consequences for morbidity or mortality of certain diseases. FGD participants, especially in Peru indicated that they understood the need confirmatory testing and would bring their study results with them to health centers, but this concept must be tested empirically. Education to the community about RDTs must include clear messages about their limitations and the need to follow up their test at a health facility.

Our study had the following limitations. First, despite using an RDT prototype, FGDs and interviews were based on hypothetical RDT availability and community-based distribution. Respondents reported they did not use thermometers nor pregnancy tests, hence it seems unlikely the community would use RDTs rather than present to health facilities. Participants expressed enthusiasm for RDT use, but only if there was a clear benefit to them for using the RDT (i.e., speed up their follow up and treatment). Second, FGD participants in Peru were predominantly female; male opinions and perspective may not have been adequately represented. RDT users in Peru, however, are most likely to be female as they tend to be responsible for family health care. Third, five FGDs carried out in Peru were done in neighborhoods participating in dengue cohort studies that may not represent the general population. We did this, however, to contrast these results with FGD participants from neighborhoods without prior exposure to research studies. We did observe some differences between the two groups: contrary to our expectations, respondents familiar with our research projects reported preferring to wait for our research team (that visits their homes twice a week) to apply an RDT instead of doing it themselves. As with most qualitative research, the use of purposive sampling was not designed to be statistically representative of the communities. Finally, there were methodological differences by country, due to differences between the two sites that needed to be explored based on issues that emerged; hence, we could not make universal comparisons (i.e. in Cambodia, FGDs did not include video instructions or participants testing the RDTs themselves).

### Conclusions

Both community and health professional focus group participants expressed enthusiastic support for at-home use of the febrile diagnostic device in Iquitos, Peru and Phnom Penh, Cambodia, as long as the use was associated with a clear benefit: faster or optimized access to health care. With simplified instructions, thorough education and awareness campaigns, and strong links to follow-up, such a device has the potential to diagnose and treat infected patients more quickly and accurately, and to be implemented in targeted surveillance and outbreak response–but numerous challenges need to be discussed and addressed. Our study shows that formative research is necessary before introduction of any such device to ensure that instructional methods for use of the device are locally appropriate, integration into local health systems is acceptable and feasible, and designed for each health infrastructure. Our findings provide support for fast tracking development, testing, and implementation of home-based rapid diagnostic devices as soon as their development is completed as they have the potential to identify outbreaks of febrile and potentially other highly contagious viral diseases.
